# Factors influencing the implementation of TB screening among PLHIV in selected HIV clinics in Ghana: a qualitative study

**DOI:** 10.1186/s12913-022-08295-6

**Published:** 2022-07-11

**Authors:** Solomon A. Narh-Bana, Mary Kawonga, Selase Adjoa Odopey, Frank Bonsu, Latifat Ibisomi, Tobias F. Chirwa

**Affiliations:** 1 School of Public Health, Faculty of Health Sciences, University of the Witwatersrand, Johannesburg, South Africa; 2 Dodowa Health Research Centre, Research and Development Division, Ghana Health Service, Dodowa, Ghana; 3 Department of Community Health, Charlotte Maxeke Johannesburg Academic Hospital, Johannesburg, South Africa; 4 National TB Control Programme, Accra, Ghana; 5 Nigerian Institute of Medical Research, Yaba, Lagos, Nigeria

**Keywords:** Barriers, Enablers, Factors, TB screening, PLHIV, Ghana

## Abstract

**Background:**

Decreasing the burden of Tuberculosis (TB) among PLHIV through TB screening is an effective intervention recommended by the World Health Organization (WHO). However, after over a decade of implementation in Ghana, the intervention does not realize the expected outcomes. It is also not well understood whether this lack of success is due to implementation barriers. Our study, therefore, sought to examine the factors influencing the implementation of the intervention among people living with HIV (PLHIV) attending HIV clinics at district hospitals in Ghana.

**Methods:**

This was a qualitative study conducted from 6th to 31 May 2019 in three regions of Ghana. We conducted 17 in-depth interviews (IDIs – comprising two regional, six districts and nine facility TB/HIV coordinators) and eight focus group discussions (FGD – consisting of a total of 65 participants) with HIV care providers. The Consolidated Framework for Implementation Research (CFIR) guided the design of interview guides, data collection and analysis. All responses were digitally audio-recorded and transcribed verbatim for coding and analysis using the Framework Approach. Participants consented to the interview and recording.

**Results:**

The main barriers to TB screening relate to the low commitment of the implementers to screen for TB and limited facility infrastructure for the screening activities. Facilitators of TB screening include (1) ease in TB screening, (2) good communication and referral channels, (3) effective goals and feedback mechanisms, (4) health workers recognizing the need for the intervention and (5) the role of chemical sellers.

**Conclusions:**

Key barriers and facilitators to the intervention are revealed. The study has shown that there is a need to increase HIV care providers and institutional commitment towards TB screening interventions. In addition, cost issues need to be assessed as they are drivers of sustainability. Our study also advances the field of implementation science through CFIR to better understand the factors influencing the implementation.

**Supplementary Information:**

The online version contains supplementary material available at 10.1186/s12913-022-08295-6.

## Background

Even though much progress has been made in the fight against Tuberculosis (TB) and HIV co-infection globally, they continue to be the main public health threats and health systems concerns in many low- and middle-income countries [1, 2]. The World Health Organization (WHO) global report on TB kept recognizing TB as the main cause of ill-health and death among people living with HIV (PLHIV) worldwide, and especially in resource-challenged settings [3–5]. In fact, in 2018, about 17% (251,000/1,491,000) of TB related deaths were in PLHIV [6]. Notwithstanding, globally, the TB deaths among PLHIV had declined over time, by 44%, from 534,000 in 2000 to 300,000 in 2017 [7]. According to the same report, about 51% of reported TB cases were from PLHIV [7]. The impact of these two diseases has been immense on the performance of health systems in poorly resourced countries and led to a reduction in global targets [8], particularly in sub-Saharan Africa [9].

Early diagnosis and effective treatment of TB cases in PLHIV can avert many deaths [7, 10, 11]. To end the dual epidemics of TB and AIDS by 2030, ensuring people are healthy and supporting the well-being of all TB and HIV patients is a prominent strategy of the WHO and Joint United Nations Programme on HIV/AIDS (UNAIDS) [7]. The WHO recommends the following three policy packages to control the TB/HIV burden: *(1)* establish the mechanism for collaboration between TB and HIV program activities; *(2)* reduce the burden of TB among PLHIV, and *(3)* decrease the burden of HIV among TB clients [12]. The second package – which is the focus of this study – is made up of three intervention groups termed as the Three I’s specifically; intensify TB case finding (ICF), isoniazid preventive therapy (IPT), and infection control for TB [12, 13]. Among these interventions, the WHO recognized ICF, done through TB screening among PLHIV, as effective in reducing the burden of TB among PLHIV [13, 14]. For example, in Ethiopia, TB screening effectively-identified 39% of PLHIV as presumptive TB cases, of which 6% were confirmed with Xpert machine [15]. ICF was defined as “the systematic identification of people with presumptive active TB, in a predetermined target group, using tests, examinations or other procedures that can be applied rapidly” [16]. For PLHIV, ICF would mean systematically screening for tuberculosis at every encounter with a health provider, among PLHIV, the high-risk population for HIV, or those living in mass population settings [17].

TB screening intervention ensures early detection of TB status followed by appropriate TB treatment or IPT among PLHIV [18]. Nonetheless, vast and persistent gaps exist in the detection and treatment of TB, including in PLHIV, especially in Sub-Saharan Africa [7]. For example, in 2017, only 64% of the estimated 10 million TB cases expected were reported, and most of the bottlenecks in detection and subsequent treatment of TB exist in the WHO Africa region where the burden of HIV co-infected with TB is highest [7].

Ghana adopted and implemented the TB screening intervention to reduce the burden of TB among PLHIV [19]. In the 2015 global TB report, Ghana was listed among the forty-one countries with TB/HIV co-infection burden with the highest estimated numbers of TB incidences that accounted for 97% of the global total [20]. In 2017, Ghana recorded an estimated TB incidence of 152 cases TB mortality of 36 per 100,000 population [21]. Also, in the same year, about 21% diagnosed with TB had HIV [21]. Ghana started the TB screening of PLHIV in 2007, with no plans to implement the TPT. The policy was revised in 2014 but contained no plans to begin the implementation of the TPT among HIV clients who were confirmed of not having TB. However, in 2018 the NTP came out with TPT initiation plan and policy for the country at all HIV clinics. The intervention in Ghana is such that all PLHIV attending HIV clinics must routinely be screened for TB with the aid of a simple questionnaire approved by WHO. The WHO-approved four-symptom questionnaire asks PLHIV for the presence of cough of any duration, fever, weight loss, and night sweats [22]. The presence of one or more symptoms means a positive screening, which requires further TB confirmatory testing through Xpert MTB/RIF (*Cepheid, CA, USA*) [23–25] and other possible tests, including sputum smear microscopy, culture for *Mycobacterium tuberculosis* and chest X-ray [26, 27]. Those with confirmed TB are put on appropriate TB treatment, while those without TB may be initiated on IPT when available [28].

A review of literature on program indicators for Ghana shows that during the early stages (from 2008 to 2012) of the implementation of the TB screening intervention, a high proportion of PLHIV (70–96%) were screened for TB at HIV care clinics [29]. Unfortunately, this observed success has not been sustained as programme data further revealed that in the years 2013 and 2014, approximately 20% [29] and 59% [30] of PLHIV, respectively, were screened for TB as part of care. Moreover, between 2010 and 2014, no clear screening target was set. However, in the 2014 revised guideline, new TB screening targets were set at 56% for 2015, and progressively increasing the target to 80% in 2018 and further progress to 90% in 2020 [29]. But in 2018 and 2020, 40 and 44% TB screening coverage were achieved, respectively, far below the annual target. Such slow and fluctuating progress and not sustaining coverage in Ghana is unknown since no research has been conducted to that effect. Implementation science is crucial in identifying and addressing implementation gaps when evidence-based interventions are delivered into routine practice. Such identified gaps can inform appropriate strategies for successful implementation. Despite over a decade that the TB screening intervention has been implemented in Ghana, little information is known about factors that influence its implementation. Hence, the current research has the potential of addressing this gap. To the best of our knowledge, this marks the first study to examine the factors (barriers and facilitators) influencing the implementation of TB screening among PLHIV with the WHO approved questionnaire.

Our study was guided entirely by the Consolidated Framework for Implementation Research (CFIR) [31], one of the many determinant frameworks available for understanding contextual and other factors influencing the implementation of intervention of proven effectiveness [32]. It is a comprehensive framework in implementation science [31]. It was developed to guide the successful implementation of interventions through ascertaining factors that may influence the implementation of the TB intervention in a given setting. This could help identify potential strategies for overcoming any challenges before the implementation.

The CFIR framework, was developed in the United States and has been applied to interventions to manage weight among Veteran’s Affairs in the United States, and promote healthy eating and physical activity in Austria. To our knowledge, the CFIR has not previously been applied to TB screening services among PLHIV in either high or low and middle-income country (LMIC) countries including Ghana. However, the framework has successfully been used in low- and middle –come countries to guide design and data collection [33, 34], data analysis [33–38], and contextualize findings [38–40]. In Ghana for instance, the CFIR was used in a qualitative study to guide data analysis [41]. Another study [42] conducted to assess nurses’ perception, facilitators and barriers of an intervention delivery in the health facility used the framework appropriately to guide the development of the interview guides and data analysis.

The CFIR comprises five domains: intervention characteristics, outer setting, inner setting, characteristics of individuals, and implementation process. The process domain entails planning, engaging, executing, and reflecting and evaluation that can only be ascertained in either a spiral or incremental approach to the implementation process [31], e.g., using the plan-do-study-act method through observation. Considering the focus of our study, we are therefore unable to include the process domain. We, therefore, chose the domains which were relevant and feasible to assess in the context of our study, defined as follows [31, 43–45]:

1. *Intervention characteristics*: features of the TB screening intervention that may influence its effective implementation in Ghana. It comprises its perceived internal or external origin, evidence quality and strength, relative advantage, complexity and cost.
2. *Outer setting*: concerns with external influences (factors outside the HIV care clinics) on implementation of TB screening comprising availability of resources, incentives, communication and networking among HIV care clinics in Ghana.
3. *Inner setting*: relates to characteristics within the health facilities delivering the intervention. These include stakeholders, compatibility and relative priority of the TB screening intervention, goal-setting and feedback and the implementation climate.
4. *Individuals’ characteristics*: concerns with health care providers and TB/HIV coordinators personal factors that may affect the implementation of the TB screening intervention. It consisted of health care providers’ knowledge, beliefs, age, and years of practising and professional background.

This article with guidance from CFIR aims to examine the factors influencing the implementation of the TB screening intervention among PLHIV attending HIV clinics at district hospitals across three regions in Ghana.

## Methods

### Aim and design

We conducted an exploratory and purely qualitative study using in-depth interviews (IDIs) and focus group discussions (FGDs) on understanding the barriers and facilitators of implementing the TB screening intervention among PLHIV attending HIV clinics in the Northern, Eastern and Central regions of Ghana. The CFIR [31] guided the design of interview guides, data collection and analysis and reported in line with the Standard for Reporting Qualitative Research (SRQR) [46].

### Setting of the study and the intervention

This study is part of a more extensive study conducted in Ghana. Ghana has ten traditionally administrative regions. The regions are subdivided into 216 districts, with most districts having a district hospital. At the time of conduct of the study, there were 144 district hospitals across the country, out of which 100 had HIV clinics. The national TB programme’s plan was to initiate IPT among PLHIV with a negative TB screen at HIV clinics in Ghana. Therefore, 27 district hospitals were earmarked to start the implementation, after which it would be scaled up to other district hospitals [19] as described and published elsewhere [47, 48]. Therefore, the main study was conducted in the 27 district hospitals across all the regions. Also, the ten regions were grouped into three ecological zones (Savannah, Forest and Coastal). One region was randomly selected from each zone to conduct this study. Therefore, this study was conducted in the three randomly chosen regions and their respective districts and district hospitals that were part of the extensive study (the 27 district hospitals).

The National Tuberculosis Programme (NTP) and the National HIV/AIDS Control Programme (NACP) jointly designed the TB screening intervention and implemented it through a cascading process at the national, regional, district, and facility levels and coordinated by TB/HIV collaborative committees. At each level, a TB/HIV coordinator was appointed “to be responsible for the day to day running of programme implementation and oversight of TB/HIV collaborative activities” [29]. At the same time, the healthcare providers at the HIV clinics are the only staff mandated to deliver the intervention to PLHIV.

Screening tools, algorithms, programme guidelines, training manuals reflecting TB/HIV co-infection managements are in place to facilitate the implementation. The Screening tools are deployed with capacity building of all clinic staff and quality of intervention is monitored through scheduled visit by National supervisors. TB counselling, health education, and infection control are frequently follow on activities per the national guideline recommendations. The cost of all the services are borne by Government of Ghana. The facilities assure quality implementation through review meetings. The intervention is being implemented ART Centre or HIV clinics in Ghana.

According to the policy, at every HIV client visit to the facility, well-trained health staff must screen the client using TB symptom screening tool. For those who respond “yes” to any of the signs and symptoms (i.e. cough of any duration, night sweat, chest pains, weight loss and fever) should be referred for an Xpert/MTB/RIF test. If MTB is detected in the client, staff must initiate anti TB therapy if Rifampicin is sensitive or if client is Rifampicin resistant refer to regional MDR TB. For clients who test negative for MTB, staff must conduct a chest x-ray if available in the facility and if chest X-ray I s abnormal refer client for further evaluation else (chest X-ray) is normal, initiate client on TPT.

For those who have no signs and symptoms after applying the symptoms screening tool. The policy requires staff to do a chest x-ray if available. If chest X-ray is normal, staff must initiate client on TPT otherwise client must be referred for an Xpert/MTB/RIF test. If MTB is detected, the client must be initiated on an anti TB therapy if Rifampicin is sensitive else refer to regional MDR TB. And finally, for clients who test negative for MTB, they must be referred to a TB expert for further evaluation.

### Characteristics of the interviewees, recruitment, and sample selection

We gathered information from TB/HIV coordinators and HIV care providers from the three randomly selected regions. We purposively selected the regional, district and facility TB/HIV coordinators to conduct IDIs to elicit information mainly at the facility level. These coordinators are public health officers and were mainly disease control officers but in their absence, nutrition officers, and health information officers were appointed to coordinate.

On other hand, HIV healthcare providers working in the HIV clinics for at least 1 year were selected to conduct FGDs to gather information at the provider level. The healthcare providers in this category included nurses, disease control officers, nutrition officers and health information officers working at the HIV clinic. Healthcare providers who did not provide direct care to PLHIV and unavailable TB/HIV coordinators at the of the data collection were excluded.

Pre-informatory letters were sent to the selected region and districts directors of health services and the facilities involved, informing them of the study. The head of HIV clinics assisted the field teams in identifying and recruiting participants for the study. Potential participants were noted and approached on the day of the data collection. Participants were recruited based on availability, especially for the FGDs and interview appointments were explicitly booked for the IDIs when an eventuality occurred. All the participants consented before participation. In all, 17 TB/HIV coordinators (two regional, six district and nine facilities) and 65 HIV healthcare providers were included.

### Data collection

IDI and FGD guides were developed based on the four domains in the CFIR framework that we had identified to apply to our study. The IDI guide focused on the characteristics of individuals’ domains which relates more to the provider level. The FGD guide also concentrated on the inner setting domain, which relates to the facility level. In addition, both guides also covered the intervention characteristics and outer setting domains. Fact-to-face IDIs were conducted with TB/HIV coordinators and FGDs with HIV healthcare providers.

Three field teams (four members per team) worked in one region each to collect data for this study. The teams had five-days of practical training, including role-play on the IDI and FGD guides. The IDIs and FGDs were conducted in English and audio-recorded using digital voice recorders under the permission and consent of the participants. The FGDs were between 7 and 10 participants, depending on availability. Interviews and discussions were held in isolated, convenient and conducive places to ensure privacy. Interview recording times ranged from about 45 minutes to about 70 minutes. Data collection was from 6th to 31st May 2019.

The audio-recorded interviews and discussions were transcribed verbatim for analysis. SNB listened to the audiotapes, compared them with the transcripts and edited them when needed to ensure the transcripts had the same information as the audiotapes. The audio recording was also used to fill in gaps in the notes. SAO reviewed and imported the transcripts into MaxQDA Analytics Pro Software 2020 for coding based on the CFIR. To ensure anonymity of responses from study participants, unique numbers were assigned to participants and used when presenting quotes under the result section.

### Data analysis

The study’s data was analysed by SNB and SAO using the Framework Approach [49], which applies both inductive and deductive methods. The framework approach involves six iterative steps being (1) familiarisation with the data, (2) generating initial codes or assignment of primary codes to the dataset to describe the content, (3) searching for patterns and codes across the different interviews, (4) codes revision, (5) codes definition and (6) producing the report through the interpretation of results was adopted. The inductive method involved steps 1 and 2, while the deductive method involved steps 3 to 6. The codes were discussed and compiled into a codebook to guide the coding and categorisation of the data. The deductive analysis approach involved coding units of data according to the CFIR, with themes categorised based on defined factors (barriers or facilitators).

On the other hand, the inductive approach was used to identify new themes or unexpected findings aside from the CFIR through coding and categorisation. The transcripts were coded in MAXQDA Analytics Pro Software 2020 using the codebook developed. During the coding process, the emerging themes were linked to four domains of CFIR.

### Reflexibility of the data analysts

SNB and SAO who analyzed the data had no relationship with the study participants before conducting the IDIs and FGDs. Although they work with the Ghana Health Service, their work is to conduct health research. They do not work directly in the health facilities mentioned in this study and therefore they were not part of the program implementation. SNB and SAO have prior research experience in almost every part of Ghana not necessarily the health facilities in which this study was conducted.

### Ethical considerations

This study which is part of a large project obtained ethical approval from the University of the Witwatersrand, South Africa (ref: M190110) and the Ethical Review Committee of the Ghana Health Service (ref: GHS-ERC002/01/19). All procedures involving human participants performed in this study were in accordance with the ethical standards of the institutional and national research committee and with the 1964 Helsinki Declaration and its later amendments or comparable ethical standards.

## Results

### Characteristics of participants

Table 1 summarises the total number of participants recruited from the randomly selected three (Northern, Eastern and Central) regions. We conducted 17 IDIs with TB/HIV coordinators from these three regions. Additionally, we conducted eight FGDs with HIV care providers from eight IHV clinics, respectively. A total of 65 HIV healthcare providers participated in the eight FGDs.

**Table 1:** Total number of enrolled participants from the Northern, Central and Eastern regions

Participants	Type of data collection	Savannah Zone	Forest Zone	Coastal Zone	Total
Regional TB/HIV coordinators	In-depth interview (IDI)	0	1	1	2
District TB/HIV coordinators	In-depth interview (IDI)	1	2	3	6
Facility TB/HIV coordinators	In-depth interview (IDI)	2	4	3	9
HIV care providers	Focus group discussion (FGD)	2	3	3	8
HIV care providers	Number of participants for the FGD	14	29	22	65

Table 2 displays the background characteristics of the participants involved in the study. Out of the 17 IDIs conducted, two (12%) were regional, six (35%) were district, and nine (52%) were facility TB/HIV coordinators, respectively. Most of the TB/HIV coordinators who participated in the IDI were males (*n* = 11, 65%), while six (35%) were females. They age from 27 to 59 years old. Our participants have worked for between 2 to 20 years, with over 82% having tertiary, while about 17% post-secondary non-tertiary education level. On the other hand, out of the 65 FGD participants, 39 (60%) were females, and their ages ranged from 24 to 59 years old. Most of them were clinicians (nurses) and had worked in an HIV clinic for between 1 to 15 years. Among the FGD participants, about 55% had attained tertiary, 39% had post-secondary non-tertiary, and 6% had upper secondary level of education.

**Table 2:** Background characteristics of study participants

Characteristics	Number (%), median (range)	
	FGD (*n* = 65)	IDI (*n* = 17)
Sex		
Female	39 (60.0)	6 (35.3)
Male	26 (40.0)	11 (64.7)
Age (median, range)	32 (24–59)	37 (27–59)
Level of education		
Upper secondary	25 (38.5)	0
Post-secondary non-tertiary	4 (6.1)	3 (17.6)
Tertiary	36 (55.4)	14 (82.4)
Profession		
Clinician	39 (60.0)	–
Non-clinician	26 (40.0)	–
Years working (median, range)	3 (1–15)	6 (2–20)

### Factors influencing the implementation of TB screening among PLHIV

The themes emerging from our data were linked to the four domains of the CFIR (Table 3). The study has shown that barriers to TB screening include lack of commitment of health workers to screen HIV clients, limited or lack of privacy and space allocated for TB screening activities. Further, the enablers of TB screening relate to ease in TB screening, good communication, and networking between colleagues within the HIV clinic and within the facility. Other enablers included stakeholder engagements, good referral channels and health workers recognizing the need for the intervention. These are described in detail in the following section.

**Table 3:** Emerging barriers and facilitators linked to CFIR domains

Domain	Themes	
	Enablers	Barriers
**Intervention characteristics**	Ease of TB screening tool	
	Goals setting, feedback and supervision	
**Outer setting**	Good referral relationships	
	Chemical sellers key in community case finding	
**Inner setting**	Renovations and innovations	Limited or lack of privacy and inadequate space to conduct TB screening
**Individual characteristics**	Acceptability of intervention among providers	A low commitment of care providers

### Intervention characteristic

In exploring the features of the intervention that might influence the implementation of the TB screening intervention at the HIV clinic, our study found factors such as ease of the TB screening, and goal setting, feedback and supervision as facilitators.

#### Ease of TB screening

Across our in-depth interviews and focus group discussions held, it was evident that the TB screening intervention was not challenging to implement. In exploring the complexity of TB screening, all the participants in the study stated that it was not complicated.

“ … the screening itself is not complicated” – *FGD with HIV care providers group* ***7***

“The screening, for us, it is not complicated”- *IDI with Facility TB/HIV Coordinator* ***7***

Generally, the participants at the facility, district and regional levels expressed ease of using the TB screening tool and emphasized that the screening tool has all the questions a provider has to ask.

“t is not complicated because if you take a good look at the screening tool it has all the questions you need to ask the client so it makes it easy” - *FGD with HIV care providers group* ***5***

“ … It [TB screening] is not complicated. It is very simple” - *IDI with Regional TB Coordinator 1*

#### Goals setting, feedback and supervision

National targets drive TB screening. However, the providers set facility-specific annual targets of cases to screen and detect from the interviews and discussions. Implementation of the intervention is monitored through monthly or quarterly reports from the providers to the district coordinators. In addition to the reports, the coordinators embark on quarterly field visits. During such visits, the patients are interviewed to verify the implementation of the intervention. This was perceived as a facilitator to implementation.

“We go on supportive supervision … There is also a database for reporting. Through that database, we are also able to monitor what they [providers] are doing, give feedback and discuss issues … When we go on monitoring and supervision we look at their data and interview some of the patients and they attest to the fact that they [patients] have been screened for TB and the data that they [providers] have also shown that they [providers] have been screening the patients for TB*”- IDI with Regional TB/HIV Coordinator* ***1***

District-level reports are prepared and sent to regional and national offices for dissemination and further decision making. There are also validations and peer reviews of reports. The care providers agreed that the feedback they receive “are helpful because you see where you have gotten to, and you see where your lapses are and where you have to put in more effort to improve.” – *IDI with Clinic head.*

### Outer setting

In exploring factors outside the HIV care hospitals, it emerged during our FGDs and IDIs that interactions with care providers in other facilities, referral relationships, and chemical sellers’ roles were facilitators to the TB screening intervention, as discussed in detail below.

#### Referral relationships

The providers explained that their clinics relate with or network with their colleagues in other facilities who also implement TB screening intervention among PLHIV. The networks involve liaising with them, referring cases to them, discussing complex case management, sharing resources, and attending training and workshops. The care providers added that the relationship and collaboration between the districts and their respective facilities are excellent. This was perceived as an enabler to TB screening activities.

“We have the genXpert. We are the only facility that has it in the whole Tamale Metropolis and they [other HIV clinics] all come to use it. They have our phone numbers. They call us as they come here to take their results from us … they come here, they bring their clients to us... we have a WhatsApp group so we are always in contact. So we have a network and the network is really strong and because we have each other's numbers, anytime there is a problem, it's easier for us to communicate and assist each other.” – *IDI with facility TB/HIV Coordinator 9*

"With the HIV clinic we collaborate, when you come and you have HIV, we screen you for TB, you have TB we screen you for HIV, it is like our one-stop shop. We just do everything for you but if you have to be referred, you are referred" - *IDI with Facility TB/HIV Coordinator* ***7***

#### Licenced chemical sellers key in community case finding

The technical policy and guideline for TB/HIV collaboration encourages all stakeholders working at the community level to include TB and HIV services and activities in their services. The district-level coordinators added from the interview that Licenced Chemical Sellers (LCS, over-the-counter medicine sellers) were essential stakeholders because they are the first point of call within the communities. LCS are the first point of care, especially when people experience mild symptoms of illness. Their main role as stakeholders is to refer people who they screen and have any sign or symptoms of TB to an HIV or TB clinics. During our interviews, our participants confirmed the role of these chemical sellers in TB case finding in the communities.

A coordinator stated that “when somebody is coughing, the first point of call will be the chemical seller to get some medication. So we work with them, from time to time we meet them and we discuss how they can link those cases to us so that together we help the patients” – *IDI with district TB/HIV Coordinator* ***4***

“The chemical sellers…because that is the first point of call, they are very important to case finding and screening so we always engage them.”- *IDI with district TB/HIV Coordinator* ***2***

The contribution of the LCS enables case finding and ultimately screening for TB.

### Characteristics of individuals

The characteristics of the individuals involved in the implementation were explored to identify their influence on the conduct of the TB screening intervention. As presented below, we found the acceptability of TB screening intervention among providers as facilitators and lack of commitment of care providers as a barrier that influences the implementation.

#### Acceptability of intervention among providers

The intervention is also widely accepted among care providers. In all the facilities we involved in this study, the providers acknowledge the need for TB screening among PLHIV. They added that the intervention is good and advantageous to PLHIV. Therefore, the intervention is important and needful because it will help in the early detection and treatment of new cases. These perceptions drove the health workers to perform the TB screening intervention.

“ … HIV clients are prone to get TB … so there’s a need to screen them to know their status.” – *IDI with facility TB/HIV Coordinator* ***6***

In fact, knowledge of the relevance and significance of TB screening among PLHIV was repeated by several of our participants. This has therefore enabled them carry out the screening intervention.

“Yes, there is the need because every person living with HIV must be tested for TB because if they are tested and they are active or positive, they can be put on drugs so that they are treated or they are cured on the TB” – *FGD with HIV care providers Group* ***8***

#### Low commitment of care providers

Although generally, the health workers have accepted the need and relevance of the intervention, some health workers’ attitudes towards the intervention were perceived as a barrier to implementation. Health workers carry out TB screening; therefore, their commitment or otherwise will directly affect the success of the intervention. Notably, coordinators at the facility, district and regional levels alluded to the fact that a major problem is the willingness of some facility-level care providers to implement the intervention.

“I think it’s the human factor. The will to want to implement the tool is the problem … it’s the commitment.”- *IDI with Facility TB/HIV Coordinator* ***9***

“The steps are not cumbersome but then, it is the support that is not coming from the facility level, you only ask for cough, that is the cardinal sign but then the commitment is not there on behalf of the health workers”- *IDI with Regional TB/HIV Coordinator* ***2***

Also, the infectious nature of TB discourages health workers from fully committing to the screening activities. It emerged from our analysis that the fear of possibly contracting TB when screening PLHIV in congested spaces contributed to this.

“Screening is not anything difficult but I think, because of the infectious nature of TB, most people don’t want to involve themselves in it”- *IDI with District TB/HIIV Coordinator 3*

### Inner setting

The participants highlighted internal factors such as space, privacy, renovations and innovations as key determinants to TB screening. These infrastructural and structural changes within the HIV care clinics are further elaborated on below.

#### Privacy and space

According to the participants, lack of privacy and inadequate space at the TB/HIV care units within the hospitals/clinics is a potential barrier to TB screening. Congested spaces allocated to TB screening also pose a threat to the providers’ health. Other providers mentioned that they did not have a laboratory for their collected samples. They added that these issues hindered the smooth implementation of TB screening for PLHIV. For example, during a focus group discussion, it emerged that “privacy is a paramount problem in our unit, the place is always choked.” – *FGD with HIV care providers Group* ***9***

“The unit we have for both TB and HIV clients is very small because both of them come to the same place on the same days for medication, screening and other activities … the place is even congested, their confidentiality is at stake” - *FGD with HIV Care providers group* ***3***

#### Renovations and innovations

Other providers also reported that there had been changes in some health facilities to enable TB screening activities among PLHIV. These changes included creating additional rooms for TB/HIV activities, introducing new HIV registers, electronically capturing patients’ data, and acquiring other screening equipment. The providers also explained that these alterations help in implementing the intervention.

“ … there is a new HIV register. Unlike the former one where it was not clearly [stated] in it, it was not enforced whether the HIV client has been screened for TB unless in the folder but this time around, in the folder, in the register, [and] on the e-tracker, it demands that. And that will encourage, it will push service providers to pay more attention to TB screening…” – *IDI with TB/HIV Coordinator* ***3***

Innovations such as the “… introduction of the screening tool is helping the work … the x-ray is also helping with the screening and at the lab we have the gene expect that is also helping to detect cases, so policy-wise I think that is where the nation is now, trying to use the best of equipment to get the best of result.” – *FGD with HIV care providers Group* ***6.***

## Discussion

The ultimate goal of TB case-finding and treatment is to cure the individual who has the disease, decrease its spread to other persons, mitigate death and avert disease-associated disability. Therefore, when cases are unidentified, treatment initiation is impossible. Not treating infected persons and noncompliance to TB treatment exposes the treatment cascade to failure, relapse and drug resistance with its associated complications [50]. The current study is critical as it reveals the existing factors (barriers and enablers) implementing TB screening among PLHIV in Ghana.

The emerging barriers and facilitators fall under the four domains explored in the study. Barriers to TB screening emerged from individual characteristics (i.e. low commitment of health workers) and inner setting (i.e. facility space and privacy allocated for screening activities). Facilitators of TB screening (emerging from all four domains) include (1) ease in TB screening, (2) good communication and referral channels, (3) effective goals and feedback mechanisms, (4) health workers recognizing the need for the intervention and (5) the role of chemical sellers.

In this study, the commitment of health workers towards the intervention is a barrier to implementation. In clinics where the workforce is lax and partially involved in the screening process, the outcome of the intervention is also affected. Low commitment of health workers towards TB control practices was cited by Tamir et al. (2016) as a barrier to achieving outcomes. This was purported to have been caused by many health workers’ lack of motivation and interest in the intervention. Given that one of the facilitators for TB screening in our study was recognizing the need for the intervention, incentivising health workers and continuous engagement with them through training workshops [51] is plausible to drive commitment towards effective implementation.

Quite relative to the involvement of health workers is the risk associated with screening, sputum collection and care of TB patients in congested spaces [52]. The staff-patient ratio aggravates the situation in these HIV clinics, where space and privacy are compromised because of resource constraints. In similar studies in Nigeria and China, the health workers adopted ways to keep their consulting and treatment areas well ventilated to control the spread of TB. Some health workers expressed concern over their health and safety, considering TB is contagious [53, 54]. Addressing issues of space will boost the commitment and implementation drive for TB screening activities [55].

Our study also revealed that TB screening and case finding cascade includes informal players such as chemical sellers. As noted above, chemical sellers are stakeholders in Ghana’s health system and mediate social behaviours of health [56]. This group of ‘treatment outlets’ offer treatment options to TB patients and help mainstream health workers detect cases. Therefore, there is a need to constantly engage and liaise with the chemical sellers to effectively implement TB screening through active case finding [57]. Networking and communication among health workers through sharing testing equipment and social media platforms like WhatsApp have offered collaboration opportunities for TB screening and case detection. The TB treatment chain has equally been strengthened through these internal and external support structures that the health system has. This provides an opportunity for driving implementation using these facilitators. Overall, the implementers noted that the TB screening intervention among PLHIV attending HIV clinics was important and necessary in reducing or decreasing the burden of TB in HIV clients in Ghana.

Issues of renovation, space and resource availability as well as other factors identified in this study have cost implications. Tamir et al. (2016) noted that renovations and other structural changes were not done due to financial constraints [52]. Cost is a primary driver of sustainability. Therefore, a broader dialogue on the cost of TB screening, its funding and accompanying resources is needed, especially in this era of a global Covid-19 pandemic. Resource limitations re-directed and limited donor aids will leave some interventions strained, and TB is no exception.

### Strength and limitation

Our study is the first to document the factors that influence the implementation of TB screening among HIV clients in Ghana. Also, a fundamental implementation outcome – fidelity – is unpacked from an implementer’s perspective to shed light on inherent, latent and apparent factors that determine the extent to which the TB screening intervention is carried out in HIV clinics in Ghana. Our study also utilised a combination of robust implementation frameworks to explore TB screening in Ghana holistically. This has allowed for an unconventional approach to identifying implementation bottlenecks and advantages that researchers, implementers, and policymakers can leverage to reach Ghana’s optimum TB care targets.

The main limitation of our study is that only seven providers participated in some of the FGD, which violates the minimum number required for an FGD. Our study may be at risk of social desirability bias and we did not perform participant checking to verify our interpretations of the data. Nonetheless, we found energetic interactions among participants in such discussions, which led to stimulations and providing first-hand information and new ideas from the HIV healthcare providers.

## Conclusion

Our study presents critical points for the TB/HIV community despite the above limitations. First, our work shows that although there are barriers to implementing TB screening among PLHIV in HIV clinics in Ghana, the enablers or facilitators would contribute to case identification and eventually treatment of TB among PLHIV. Second, there is evidence of an overall recognition of the need for the intervention. Third, there is also a need to: (1) strengthen commitment of health workers; (2) address space constraints at the HIV clinics (3) deliberately and constantly engage other influential stakeholders such as chemical sellers and (3) explore sustainable ways for caring for this vulnerable population beyond donor support, especially during the Covid-19 pandemic when economies are under pressure to thrive, and foreign aids are gradually diminishing or being re-directed. Finally, our study showed that there are more facilitators to leverage to achieve successful implementation and optimal coverage of TB screening among PLHIV in Ghana.

This study may also provide relevant insights to engage with Ghana Health Services in developing the intervention to ensure outcomes from this study are utilised.

## Supplementary Information

**Additional file 1.**

## Data Availability

The interview and discussion guides used to collect data for this study has been uploaded under supplementary file with the filename “additional file 1.docx”.

## Abbreviations

HIV: Human Immunodeficiency Viruses
PLHIV: People living with human immunodeficiency viruses
TB: Tuberculosis
ART: Antiretroviral therapy
IDI: In-depth Interview
FGD: Focus Group Discussion
T&T: Travel and Transport
CWC: Child welfare clinic
ANC: Antenatal Care
