# Supplementary material for: Factors influencing the implementation of TB screening among PLHIV in selected HIV clinics in Ghana: a qualitative study
Source: BMC Health Serv Res. 2022 Jul 11;22:898. doi: 10.1186/s12913-022-08295-6 (PMC9272598; doi:10.1186/s12913-022-08295-6)
Supplement: Supplementary file 1 — Additional file 1. [file 12913_2022_8295_MOESM1_ESM.docx]

**UNIVERSITY OF THE WITWATERSRAND**

**FACULTY OF HEALTH SCIENCES, SCHOOL OF PUBLIC HEALTH**

Study title: ASSESSING THE IMPLEMENTATION FIDELITY AND DETERMINANTS: A CASE OF ACTIVE TB CASE-FINDING AND IPT INITIATION AMONG PLHIV ATTENDING HIV CLINICS IN GHANA

For IDI with facility, district and regional TB/HIV coordinators

1. **Background information**

- Start time
- Date of interview
- Respondent ID (participant code):
- Respondent type: (interview with…)
- Respondent sex:
- How old are you?
- What is your educational level?
- What is your occupation / position?
- How long have you been working in this unit/facility/capacity?
- Please tell me about your main roles and responsibilities

1. **Intervention Characteristics**
   1. **Evidence Strength and Quality**
2. What kind of information or evidence are you aware of that shows whether or not the TB screening among PLHIV is working in your facility?
   - What are the sources of your information or evidence?
3. In a healthcare setting, some stakeholders may include where and how interventions get implemented. Who do you think are the influential stakeholders when it comes to implementation of the TB/HIV collaborative activities - particularly TB screening and IPT in PLHIV?
   - **Probes:** Stakeholders in this facility?

Stakeholders outside this facility?

In what ways do these stakeholders influence implementation?

**b. Relative Advantage**

1. How does the intervention for TB screening and IPT initiation among PLHIV compare to other similar existing programs in this facility/district/region

Probe for:

- - Any advantages compared to other existing programs?
  - Any disadvantages compared to other existing programs?

**c. Complexity**

1. Would you say the TB screening, is complicated? Please explain your response – in what ways would you say it is complicated / not?
   - **Probe**: what about in terms of: the duration, scope, intricacy, number of steps involved? Does the intervention reflects a clear departure from previous practices (HIV care?
2. What do you think about the intervention of giving IPT for PLHIV? Would you say it is complicated? Please explain– in what ways it is complicated / not?
   - **Probe**: what about in terms of: the duration, scope, intricacy, number of steps involved? Does the intervention reflects a clear departure from previous practices (HIV care and TB screening only?

**d. Cost**

1. What costs are incurred by this facility / district / region that you know in the implementation of the TB screening and IPT intervention among PLHIV?
2. **Outer setting**
   1. **Cosmopolitanism**
3. Do providers in this HIV care clinic relate with or network with their colleagues outside your facility who are also implementing TB screening and IPT intervention among PLHIV?
   - Probe if no, why?
4. If yes, how do providers in this HIV care clinic relate with or network with their colleagues outside your facility who are also implementing TB screening and IPT intervention among PLHIV?

- Probes: Any information exchange? What kind of information? How?

Ability to attend conferences and trainings?

To what extent facility encourages providers to network with colleagues outside?

1. **Inner setting**
   1. **Structural characteristics**
2. Please tell me about infrastructure in your facility?
   - ***Probe***: Social architecture (design of the place encourages access), age, maturity, size, or physical layout.

How infrastructure may hinder or facilitate implementation of the TB screening among PLHIV?

1. Please tell me about any structural changes this facility/ district/ region made towards sustaining the TB screening among PLHIV?
   - ***Probe for*** changes in scope of practice? Changes in formal policies? Changes in information systems or electronic records systems? Other structural changes?
   - How did you work around these changes?
   1. **Networks & Communications**
2. Please describe working relationships amongst colleagues in this facility?
   - Between colleagues within the HIV care unit/clinic?
   - Between colleagues in the HIV care unit and other units?
   - Are meetings, such as staff meetings, held in this HIV care unit?
   - Who typically attends?
   - What proportion of staff typically attend?
   - How often are the meetings held?
   - What is the typical agenda?
   - How helpful are these meetings towards implementation?
3. How do you usually find out about new information, such as new initiatives, accomplishments, issues, new staff, and staff departures?
4. When you need to get something done or to solve a problem, who are your "go-to" people?
5. **Implementation Climate**
   1. **Tension for Change**
6. Do you see a strong need for this TB screening among PLHIV intervention?
   - If yes, why or if no, why not?
   - Do others see a need for the intervention?
   - How important is this intervention to meet the needs of the individuals served by the facility / district / region?
   - How do people within your facility / district health level / regional health level feel about current programs/practices/process that are related to the intervention?
   1. **Relative Priority**
7. What kinds of high-priority activities are happening in your facility / district / region?
   - How, if at all, is this intervention on TB screening in PLHIV conflicting with these priority activities?
   1. **Organizational Incentives & Rewards**
8. What kinds of incentives are there to help ensure that the implementation of the intervention is successful?
   - What is your motivation for wanting to help ensure the implementation is successful?
   1. **Goals & Feedback**
9. Have you/your unit/your organization set goals related to the implementation of the intervention?

[If yes] What are the goals?

- To what extent are organizational goals monitored for progress?
  - Can you give an example of monitoring in terms of the type of information, who is informed, and how?
  - Do you get any feedback reports about your work?
  - What do they look like? Content, mode, form?
  - How often do you get them? Where do they come from?
  - How helpful are those reports? How can they be improved?

**Is there any other information you would like to share?**

**Thank you for the information and your time! Time interview ended**

**UNIVERSITY OF THE WITWATERSRAND**

**FACULTY OF HEALTH SCIENCES, SCHOOL OF PUBLIC HEALTH**

Study title: ASSESSING THE IMPLEMENTATION FIDELITY AND DETERMINANTS: A CASE OF ACTIVE TB CASE-FINDING AND IPT INITIATION AMONG PLHIV ATTENDING HIV CLINICS IN GHANA

For FGD with HIV care providers

- - 1. Start time
    2. Date of interview

1. **Background information *(Please use the FGD recruitment form to capture section A)***
   - 1. Respondent ID (participant code):
     2. Respondent sex:
     3. How old are you?
     4. What is your educational level?
     5. What is your occupation / position?
     6. How long have you been working in this unit/facility/capacity?

*Instruction for the moderator: Set rules for the discussion and encourage participants to speak louder*

1. Please tell me about your main roles and responsibilities
2. What do you know about the TB screening among PLHIV intervention?
3. How confident do you as HIV care providers feel about performing screening for TB among PLHIV?
4. What gives you that level of confidence (or lack of confidence)?
5. **Intervention Characteristics**
   1. **Evidence, Strength and Quality**
6. What kind of information or evidence are you aware of that shows whether or not the TB screening and IPT initiation among PLHIV is working in your facility?
   - What are the sources of your information or evidence?
7. In a healthcare setting, some stakeholders may include where and how interventions get implemented. Who do you think are the influential stakeholders when it comes to implementation of the TB/HIV collaborative activities - particularly TB screening and IPT initiation in PLHIV?
   1. **Probes:** Stakeholders in this facility?

Stakeholders outside this facility?

In what ways do these stakeholders influence implementation?

**b. Relative Advantage**

1. What are some of the existing programs or interventions on going in this facility?
2. How does the intervention for TB screening and IPT initiation among PLHIV compare to these other existing programs in your setting?
   1. Any advantages compared to other existing programs?
   2. Any disadvantages compared to other existing programs?

**c. Complexity**

1. Would you say the TB screening, is complicated? Please explain your response – in what ways would you say it is complicated / not?
   1. **Probe**: what about in terms of: the duration, scope, intricacy, number of steps involved? Does the intervention reflects a clear departure from previous practices (HIV care?
2. What do you think about the intervention of giving IPT for PLHIV? Would you say it is complicated? Please explain– in what ways it is complicated / not?
   1. **Probe**: what about in terms of: the duration, scope, intricacy, number of steps involved? Does the intervention reflects a clear departure from previous practices (HIV care and TB screening only?

**d. Cost**

1. What costs are incurred by this facility / district / region that you know in the implementation of the TB screening and IPT intervention among PLHIV?
   1. **Beliefs about the intervention**
2. What are your thoughts about sustainability of the intervention in your setting?

- How could it be sustained in your setting?
- Why or why would it not be sustained?

1. **Outer setting**
   1. **Cosmopolitanism**
2. Do providers in this HIV care clinic relate with or network with their colleagues outside your facility who are also implementing TB screening and IPT intervention among PLHIV?

- Probe if no, why?

1. If yes, how do providers in this HIV care clinic relate with or network with their colleagues outside your facility who are also implementing TB screening and IPT intervention among PLHIV?

- Probes: Any information exchange? What kind of information? How?

Ability to attend conferences and trainings?

To what extent facility encourages providers to network with colleagues outside?

1. **Inner setting**
   1. **Structural characteristics**
2. Please tell me about infrastructure in your facility?
   1. ***Probe***: Social architecture (design of the place encourages access), age, maturity, size, or physical layout.

How infrastructure may hinder or facilitate implementation of the TB screening among PLHIV?

1. Please tell me about any structural changes your organisation made towards sustaining the TB screening among PLHIV?
   1. ***Probe for*** changes in scope of practice? Changes in formal policies? Changes in information systems or electronic records systems? Other structural changes?
   2. How did you work around these changes?
   3. **Networks & Communications**
2. Can you describe working relationships amongst colleagues in this facility?
   1. Between colleagues within the HIV care unit/clinic?
   2. Between colleagues in the HIV care unit and other units?
   3. Are meetings, such as staff meetings, held in this HIV care unit?
   4. Who typically attends?
   5. What proportion of staff typically attend?
   6. How often are the meetings held?
   7. What is a typical agenda?
   8. How helpful are these meetings towards implementation?
3. How do you typically find out about new information, such as new initiatives, accomplishments, issues, new staff, staff departures?
4. When you need to get something done or to solve a problem, who are your "go-to" people?
5. **Implementation Climate**
   1. **Tension for Change**
6. Do you see a strong need for this TB screening among PLHIV intervention?
   1. Why or why not?
   2. Do others see a need for the intervention?
   3. How important is this intervention to meet the needs of the individuals served by facility / district / region?
   4. How do people within your facility / district health level / regional health level feel about current programs/practices/process that are related to the intervention?
   5. **Relative Priority**
7. What kinds of high-priority activities are happening in your facility / district / region?
   1. How, if at all, is this intervention on TB screening in PLHIV conflicting with these priority activities?
   2. **Organizational Incentives & Rewards**
8. What kinds of incentives are there to help ensure that the implementation of the intervention is successful?
   1. What is your motivation for wanting to help ensure the implementation is successful?
   2. **Goals & Feedback**
9. Has/your unit/facility set goals related to the implementation of the intervention?

[If yes] What are the goals?

1. How are the goals monitored for progress?

Probe:

- 1. Do you get any feedback reports about your work?
  2. What do they look like? Content, mode, form?
  3. How often do you get them? Where do they come from
  4. How helpful are those reports? How can they be improved?

Is there any other information you would like to share?

**Thank you for participating in this discussion. Time Ended**
